# Body Fat Assessment in International Elite Soccer Referees

**DOI:** 10.3390/jfmk5020038

**Published:** 2020-06-06

**Authors:** Cristian Petri, Francesco Campa, Vitor Hugo Teixeira, Pascal Izzicupo, Giorgio Galanti, Angelo Pizzi, Georgian Badicu, Gabriele Mascherini

**Affiliations:** 1 Sports and Exercise Medicine Unit, Clinical and Experimental Department, University of Florence, 50121 Firenze, Italy; cristian.petri@unifi.it (C.P.); giorgio.galanti@unifi.it (G.G.); gabriele.mascherini@unifi.it (G.M.); 2 Department for Life Quality Studies, University of Bologna, 47921 Rimini, Italy; francesco.campa3@unibo.it; 3 Faculty of Nutrition and Food Sciences (FCNA), University of Porto, 4200-465 Porto, Portugal; vhugoteixeira@fcna.up.pt; 4 Research Centre in Physical Activity, Health and Leisure (CIAFEL), Faculty of Sports, University of Porto, 4099-002 Porto, Portugal; 5 Department of Medicine and Aging Science, “G. D’Annunzio” University of Chieti-Pescara, 66100 Chieti, Italy; pascalizzicupo@gmail.com; 6 A.I.A., Italian Referees Association, 00187 Rome, Italy; angelopizzi@alice.it; 7 Department of Physical Education and Special Motricity, Transilvania University of Brasov, 500068 Brasov, Romania

**Keywords:** anthropometry, body composition, DXA, equation, fat mass, soccer, somatotype, skinfolds

## Abstract

Soccer referees are a specific group in the sports population that are receiving increasing attention from sports scientists. A lower fat mass percentage (FM%) is a useful parameter to monitor fitness status and aerobic performance, while being able to evaluate it with a simple and quick field-based method can allow a regular assessment. The aim of this study was to provide a specific profile for referees based on morphological and body composition features while comparing the accuracy of different skinfold-based equations in estimating FM% in a cohort of soccer referees. Forty-three elite international soccer referees (age 38.8 ± 3.6 years), who participated in the 2018 Russian World Cup, underwent body composition assessments with skinfold thickness and dual-energy X-ray absorptiometry (DXA). Six equations used to derive FM% from skinfold thickness were compared with DXA measurements. The percentage of body fat estimated using DXA was 18.2 ± 4.1%, whereas skinfold-based FM% assessed from the six formulas ranged between 11.0% ± 1.7% to 15.6% ± 2.4%. Among the six equations considered, the Faulkner’s formula showed the highest correlation with FM% estimated by DXA (r = 0.77; R^2^ = 0.59 *p* < 0.001). Additionally, a new skinfold-based equation was developed: FM% = 8.386 + (0.478 × iliac crest skinfold) + (0.395 × abdominal skinfold, r = 0.78; R^2^ = 0.61; standard error of the estimate (SEE) = 2.62 %; *p* < 0.001). Due to these findings, national and international federations will now be able to perform regular body composition assessments using skinfold measurements.

## 1. Introduction

No matter the competition level, there is no official soccer game without the presence of the 23rd key element: the referee. There are more than 840,000 registered referees who arbitrate soccer games each week verifying and enforcing the rules of the game [1]. However, scientific literature on referees in relation to physiological demands, body composition, and nutrition-related aspects [2,3,4] is limited when compared to what is available on players.

Not only do elite-class soccer referees need to perform their best in perceptual-cognitive abilities and decision-making tasks [5], but they must also achieve an elevated aerobic performance similar to a midfield soccer player [6]. Although there are significant differences between games, referees cover on average a distance between 9 and 13 km per game, depending on the level of competition [3,7,8,9]. Due to the high-intensity matches in recent years [10] and as a consequence of the increase in physical demand there is a trend towards a decrease in body mass index (BMI) and body fat levels in elite referees [11]. In fact, recent studies on elite referees (FIFA) show lower levels of BMI and body fat than previously reported in Premier League referees [12,13]. 

In soccer players, body fat is generally estimated by skinfold thickness assessment, bioelectrical impedance analysis, and dual-energy X-ray absorptiometry (DXA). DXA is widely accepted as a practical method for assessing fat mass in athletes [14,15,16] and is becoming more popular in elite sports as an analysis used by both practitioners and researchers [17]. Anthropometric assessment is another popular method used to predict body fat in athletes [18,19,20]. The measurement of skinfolds is widely adopted to monitor changes in body composition due to training and/or dietary interventions. Skinfold thickness measurements have long been utilized to predict body fat and over the years multiple equations have been developed for this purpose [21,22,23,24,25,26]. However, to the best of the authors’ knowledge, there are no studies comparing results obtained by skinfold-based measurements and DXA in soccer referees. 

This study was designed to present body composition features of soccer referees called for the Russian World Cup in 2018 with two main aims. The first purpose was to compare different equations to estimate fat mass from skinfold assessment with data derived from DXA. The second aim was to develop a prediction equation for estimating the percentage of fat mass (FM%) based on anthropometric variables specific for this cohort.

## 2. Materials and Methods 

### 2.1. Study Population

Using a cross-sectional design, 43 elite international soccer referees (age 38.8 ± 3.6 years) from 6 confederations were enrolled in the study during a seminar held in the Federal Technical Center of Coverciano (Italy) in April 2018, during the competitive season. They were classified as elite because they were either registered at the maximum level in the athletic federation or have received financial support for their dedication to training and games. The study was designed and conducted in accordance with the Helsinki Declaration. The ethics committee of the Italian football association approved this study and all the participants signed written informed consent prior to their inclusion in the study (approval code: 03032018).

### 2.2. Procedures

Participants underwent body composition assessments early in the morning, in an overnight-fasted state, and at least 12 h postexercise, with no long trips during the previous day. Further, the consumption of alcohol and stimulant beverages were not allowed for at least 15 h prior to testing.

The methodology used for the assessment of body composition was in accordance with our previous studies [26,27], using the integration of anthropometry, skinfold thickness, and DXA. Anthropometric measurements were taken following the protocol of The International Society for the Advancement of Kinanthropometry (ISAK) [28] by the same researcher (an ISAK level anthropometrist), whose technical error was 5% and 1.5% for skinfolds and all other measurements, respectively. Height (m) and body weight (kg) were measured to the nearest 0.1 cm and 0.01 kg, respectively, using a high-precision mechanical scale (SECA, Basel, Switzerland). BMI was calculated using the formula body mass/height^2^ (kg/m^2^). Biceps girth, waist girth, and hip girth (cm) were measured with a narrow, metallic, and inextensible measuring tape (Lufkin^®^ model W606PM, London, UK; precision = 1 mm). Skinfolds were measured with a skinfold caliper (Holtain Ltd., Crymych, UK; precision = 0.2 mm) at nine anatomical sites (triceps, subscapular, biceps, iliac crest, supraspinal, pectoral, abdominal, thigh, and calf). Humerus and femur breadths were measured to the nearest 0.1 cm with a sliding caliper (GMP, Zürich, Switzerland). Somatotype was calculated according to the Heath-Carter method [29].

Body density was calculated from the Siri equation [30] adapted for age [31], which was then used to estimate FM%. The following six skinfold-based equations were used to estimate FM%:

- Yushaz [21];
- Faulkner [22];
- Eston et al. [24];
- Durnin and Womersley [23];
- Reilly et al. [25];
- Suarez et al. [26].

Additionally, the average FM% measured by all of these equations was considered. Fat-Free Mass (FFM) was calculated subtracting fat mass from body weight.

A DXA scanner (Hologic QDR Series, Delphi A model, Bedford, MA, USA) with Hologic APEX 13.3:3 software version, was used to estimate FM%. The instrument was calibrated with phantoms as per the manufacturer’s guidelines each day prior to measurements. Participants assumed a stationary supine position on the scanning table. All scanning and analyses were performed by the same technician to ensure consistency and in accordance with standardized testing protocols recognized as best practice [17,32,33].

### 2.3. Statistical Analysis

Data were expressed as mean ± standard deviations (SD) and normality of distribution of the data was verified by the Kolmogorov–Smirnov test. Comparisons between FM% calculated with the different formulas and those measured by DXA were made using linear regression analysis, as well as between the sum of skinfold measurements with FM% obtained by DXA.

Given the fact that different ethnic groups participated, the effect of ethnicity on FM% was tested using the Kruskal–Wallis test. No interactions were found between ethnicity and other independent variables; therefore, we used the whole sample in the model development. The ability of the following variables (age, stature, weight, and skinfolds) in predicting FM% in the international soccer referees was assessed using stepwise regression analysis. During model development, normality of residuals and homogeneity of variance were tested. Significance at *p* ≤ 0.05 was established as the criterion for inclusion of a predictor whereas removal criteria were set at *p* ≤ 0.1. If more than one variable remained in the model, and to assess multicollinearity, a variance inflation factor (threshold as 5) was calculated for each independent variable. The data were analyzed using the statistical package IBM SPSS Statistics (version 13.0) for Windows. (SPSS Inc., Chicago, IL, USA). 

## 3. Results

**Figure 1:**
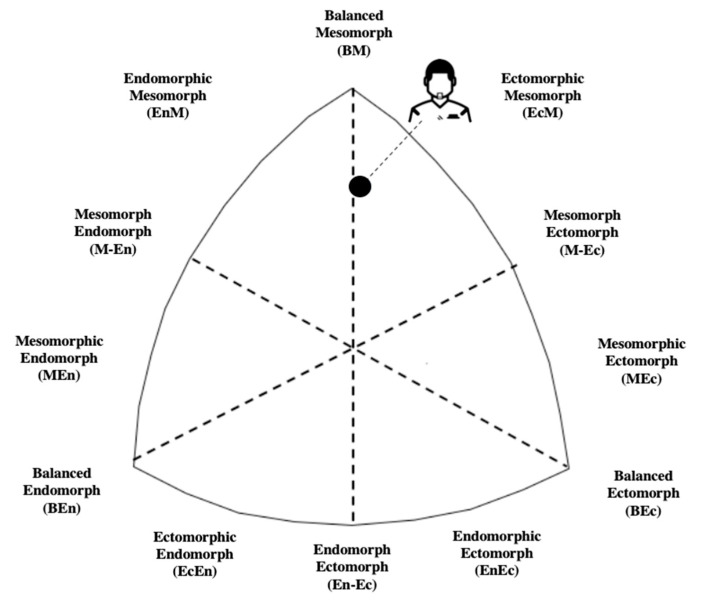
Representation of the somatotype of the International elite male soccer referees.

General and anthropometric characteristics and descriptive values of FM% estimated from DXA and skinfold-based equations are shown in Table 1. The referees showed an average balanced mesomorph somatotype, characterized by a dominant mesomorph component and similar values between endomorph and ectomorph components (no more than a difference of 0.5 units, Figure 1).

Correlation coefficients and level of significant differences between FM% with DXA and other practical estimates in the soccer referees are shown in Table 2. Given that no difference between ethnic groups in FM% was found (*p* = 0.241), all the values were presented together.

All FM% values obtained by the skinfold-based equations showed large to very large positive correlations (r from 0.60 to 0.78) to those measured by DXA. The FM% estimated from all of the equations showed significant differences (*p* < 0.001) in comparison to the DXA results. The sum of skinfold measurements showed moderate to very large positive correlations with FM% obtained by DXA, except for the sum of two skinfold measurements. Relationships between DXA-derived FM% and skinfold thicknesses of different anatomical sites are shown in Table 3. The vast majority of skinfold measurements cited showed moderate to very large positive relationships with FM% (r from 0.57 to 0.71), except biceps, pectoral, anterior thigh, and medial calf.

**Figure 2:**
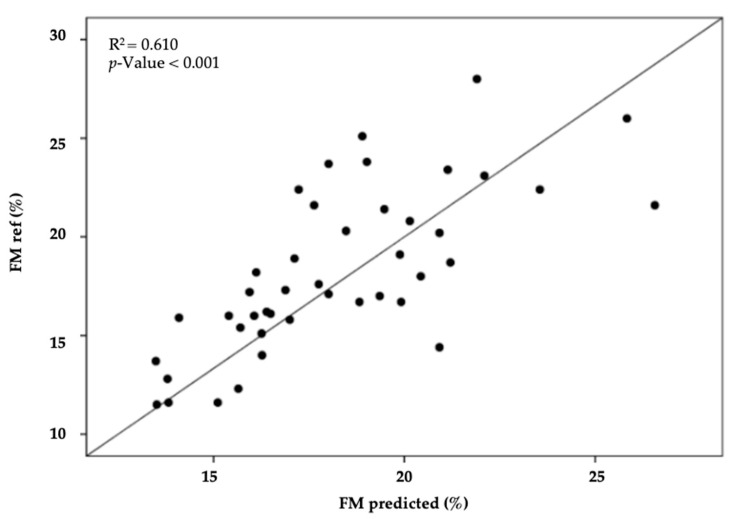
The scatter plot illustrates the results of the regression analysis for FM% obtained by the reference method (DXA) and predicted by the equation developed in the sample of international soccer referees.

Table 4 shows the skinfold-based model for FM% generated for the international soccer referees. Only variables contributing as significant predictors using backward stepwise approach were used in the model. The final prediction model included: FM% = 8.386 + (0.478 × iliac crest skinfold) + (0.395 × abdominal skinfold, r = 0.781; R^2^ = 0.610; SEE = 2.62 %; *p* < 0.001, Table 4; Figure 2). 

## 4. Discussion

This study compared six skinfold-based equations showing the different results in FM% estimation in international elite soccer referees, using DXA as a reference method. Additionally, our study is the first to have provided a specific equation for this particular sample group, as well as descriptive body composition parameters. Our results highlight that the sum of skinfold thickness measurements taken in seven (triceps, subscapular, iliac crest, supraspinal, abdominal, anterior thigh, and medial calf) or in nine (biceps, triceps, subscapular, iliac crest, supraspinal, pectoral, abdominal, anterior thigh, and medial calf) sites show a high association with the FM% estimated with DXA. Secondly, among the equations considered, that of Faulkner [22] showed the best sensitivity in assessing FM% in the international elite soccer referees. Lastly, a new equation based on the anthropometric measurements taken on the sample group was proposed. 

While excessive FM% may affect performance, body composition is an aspect of considerable interest to scientists, athletes, and coaches [34]. Typical FM% values (ranging from 5% to 19%) reported in male athletes depends on the sport, playing position, and methodology used for the assessment [35,36,37]. In particular, male soccer players show a percentage of fat mass ranging between 11.7–13.7% [20,38]. Furthermore, the tested referees in this study showed a balanced mesomorph somatotype. 

Similarly, high-level soccer players are characterized by a balanced mesomorph morphology but their somatotype can also be endomorphic mesomorph, ectomorphic mesomorph, mesomorph ectomoprh, and mesomorphic ectomorph [39]; in all these cases, the dominant component is the mesomorphy, but there is a different balance between endomorphy and ectomorphy, probably due to the different roles of the game. 

Our results showed substantial discrepancies in FM% prediction depending on the method plied. Therefore, care must be taken when feedback on FM% is provided to soccer referees since values are likely to be method dependent. With the exception of FM% obtained using Suarez’s equation [26] or estimated by DXA, most of the data found was within the range described in the literature. As opposed to Faulkner’s equation [22], the new formula suggested appears to be a simpler and faster alternative as it is specific to soccer referees. Furthermore, the use of only two skinfold sites, provides an advantage in the field-based assessment of FM%, representing a more efficient use of time.

Contrary to previous studies of elite soccer players [25,26], data collected in the present study showed that measurements of the lower body skinfolds are not accurate when predicting FM%. In this study, thigh skinfold thickness was not entered in the developed model representing strength in the new formula because it has been acknowledged that the anterior thigh skinfold is one of the least accurate sites to measure [23,24]. Furthermore, our results showed that different sums of skinfolds measured on the referees showed moderate to large correlations with DXA data, used as a criterion method. The results of the present study showed very large correlations between Σ4SKF-a, Σ4SKF-b, Σ6SKF, Σ7SKF, and Σ9SKF with FM% estimated by DXA, similar to the associations with the data found in the literature for elite soccer players [40,41]. Thus, considering the substantial differences observed between the different equations and their similar and/or lower correlations with the DXA-derived FM%, even the sum of skinfold measurements appears to be a good alternative approach in obtaining information on body fat distribution in elite soccer referees.

Some of the strengths in this study include the selection of international-level male soccer referees, all with experience in international matches. Skinfold measurement is a practical, low cost, and easily accessible alternative to more complex and expensive methods such as DXA. Consequently, the present study provides a noninvasive, cost-free, and fast tool to accurately estimate FM% in the investigated cohort. Moreover, the DXA method might not be feasible or useful when financial resources are limited or when a whole group is repeatedly measured, because up to 10 min for each person is required. 

However, the use of DXA as a reference method poses some limitations in the development and comparison of new equations for assessing fat mass. Most of the equations in the literature are based on the reference of hydrostatic weighing. This could predispose our equation to an overestimation of body fat. Additional limitations regarding sample size should be addressed. The sample consisted exclusively of male subjects thus, further studies involving elite female referees are needed. Furthermore, the lack of a validation sample did not allow the test of the performance of the new equation at a group level (e.g., analysis of the regression coefficients, line of identity, R^2^, RMSE) and at the individual level using the Bland–Altman analysis.

The practical application of this equation facilitates an increasingly accurate evaluation of the athlete. The soccer referee can be considered in all respects as an athlete, even sports research has deepened this particular population. First, the components of the physical load required during a competition [3,7,9] have been evaluated, more recently the nutritional aspects have had an increase in interest [4,12,42,43]. In this context of energy balance, the development of a new equation for the evaluation of the fat mass with a specific reference to the analyzed population allows a more accurate, reliable, and repeatable evaluation during the competitive season of the soccer referee through a field methodology.

## 5. Conclusions

All the equations investigated (Eston et al. [24], Yuhasz [21], Reilly et al. [25], Suarez et al. [26], and Durnin and Womersley [23]) showed positive correlations in comparison with DXA data. In particular, the equations developed by Faulkner [22] showed the best sensitivity in assessing FM% compared to DXA. Additionally, the sum of seven skinfolds, which included triceps, subscapular, iliac crest, supraspinal, abdominal, anterior thigh, and medial calf measurements showed a high correlation with FM% measured by DXA, representing an alternative approach in body composition assessment. Finally, this study provides a new formula for FM% estimation in international-level male soccer referees [FM% = 8.386 + (0.478 × iliac crest skinfold) + (0.395 × abdominal skinfold)].

## Figures and Tables

**Table 1:** General and anthropometric characteristics of the international-level elite referees.

Variable	Mean	SD	Minimum	Maximum
Age (year)	38.8	3.6	29.5	44.1
Body Mass (kg)	75.7	6.5	61.0	94.0
Height (m)	1.8	0.1	1.7	1.9
Body mass index (kg/m^2^)	23.1	1.3	20.6	25.8
Endomorphy	2.7	0.9	1.2	5.1
Mesomorphy	6.5	1.2	4.2	8.5
Ectomorphy	2.9	0.7	1.2	4.6
Fat Mass by DXA (%)	18.2	4.1	11.5	28.0
Fat Mass by Eston et al. [24] (%)	12.0	2.4	9.4	18.8
Fat Mass by Yuhasz [21] (%)	12.8	2.5	8.8	21.0
Fat Mass by Faulkner [22] (%)	12.7	2.1	9.2	18.5
Fat Mass by Reilly et al. [25] (%)	11.0	1.7	8.2	16.5
Fat Mass by Suarez et al. [26] (%)	15.6	2.4	12.6	24.6
Fat Mass by Durnin and Womersley [23] (%)	13.3	2.9	8.5	20.5
Fat Mass by Mean (%)	12.9	2.2	9.7	19.4
∑2sk (mm)	14.1	5.0	7.8	29.6
∑4sk-a (mm)	32.4	8.5	21.0	57.3
∑4sk-b (mm)	38.6	11.4	21.6	75.6
∑5sk (mm)	41.7	10.7	27.9	74.5
∑6sk (mm)	59.6	16.4	34.0	112.6
∑7sk (mm)	62.9	16.6	38.9	116.8
∑9sk (mm)	73.7	18.2	46.5	130.8

Abbreviations: Σ2sk = anterior thigh, medial calf; Σ4sk-a = biceps, triceps, subscapular, iliac crest; Σ4sk-b = triceps, abdominal; anterior thigh, medial calf; Σ5sk = biceps, triceps, subscapular, iliac crest, anterior thigh; Σ6sk = triceps, subscapular, iliac crest, abdominal, anterior thigh, medial calf; Σ7sk = triceps, subscapular, iliac crest, supraspinal, abdominal, anterior thigh, medial calf; Σ9sk = biceps, triceps, subscapular, iliac crest, supraspinal, pectoral, abdominal, anterior thigh, medial calf.

**Table 2:** Correlation coefficients and level of significance between fat mass percentage (FM%) estimated by dual-energy X-ray absorptiometry (DXA) with the sum of skinfold measurements, and FM% obtained from skinfold-based equations.

Variable	r	R^2^	*p*-Value
∑2sk	0.41	0.165	0.007
∑4sk	0.75	0.559	<0.001
∑4sk	0.70	0.491	<0.001
∑5sk	0.72	0.519	<0.001
∑6sk	0.76	0.585	<0.001
∑7sk	0.77	0.588	<0.001
∑9sk	0.76	0.581	<0.001
Fat Mass by Eston et al. [24]	0.60	0.363	<0.001
Fat Mass by Yuhasz [21]	0.76	0.585	<0.001
Fat Mass by Faulkner [22]	0.77	0.598	<0.001
Fat Mass by Reilly et al. [25]	0.71	0.497	<0.001
Fat Mass by Suarez et al. [26]	0.74	0.549	<0.001
Fat Mass by Durnin and Womersley [23]	0.76	0.580	<0.001
Fat Mass by Mean	0.78	0.606	<0.001

**Table 3:** Relationships between DXA-derived FM% and different skinfolds measured in the international-level elite referees.

Variable	r	R^2^	*p*-Value
Biceps	0.25	0.060	0.113
Triceps	0.57	0.321	<0.001
Subscapular	0.63	0.394	<0.001
Iliac crest	0.71	0.510	<0.001
Supraspinal	0.53	0.285	<0.001
Pectoral	0.02	0.001	0.885
Abdominal	0.68	0.464	<0.001
Anterior thigh	0.37	0.135	0.016
Medial calf	0.39	0.154	0.009
BMI	0.10	0.011	0.505

**Table 4:** Developed models for FM% prediction.

Variable	Coefficient	R^2^	SEE (%)
Model 1		0.510	2.90
Intercept	9.727		
Iliac crest skinfold (mm)	0.714		
Model 2		0.610	2.62
Intercept	8.386		
Iliac crest skinfold (mm)	0.478		
Abdominal skinfold (mm)	0.395		

Abbreviations: R^2^, coefficient of determination; SEE, standard error of the estimate.
